# Combination of Silver Nanoparticles and *Drosera binata* Extract as a Possible Alternative for Antibiotic Treatment of Burn Wound Infections Caused by Resistant *Staphylococcus aureus*

**DOI:** 10.1371/journal.pone.0115727

**Published:** 2014-12-31

**Authors:** Marta Krychowiak, Mariusz Grinholc, Rafal Banasiuk, Miroslawa Krauze-Baranowska, Daniel Głód, Anna Kawiak, Aleksandra Królicka

**Affiliations:** 1 Laboratory of Biologically Active Compounds, Department of Biotechnology, Intercollegiate Faculty of Biotechnology, University of Gdansk and Medical University of Gdansk, Gdansk, Poland; 2 Laboratory of Molecular Diagnostics, Department of Biotechnology, IFB UG & MUG, Gdansk, Poland; 3 Department of Pharmacognosy with Medicinal Plant Garden, Medical University of Gdansk, Gdansk, Poland; 4 Laboratory of Plant Protection and Biotechnology, Department of Biotechnology, IFB UG & MUG, Gdansk, Poland; Second University of Naples, Italy

## Abstract

*Staphylococcus aureus* is the most common infectious agent involved in the development of skin infections that are associated with antibiotic resistance, such as burn wounds. As drug resistance is a growing problem it is essential to establish novel antimicrobials. Currently, antibiotic resistance in bacteria is successfully controlled by multi-drug therapies. Here we demonstrate that secondary metabolites present in the extract obtained from *Drosera binata in vitro* cultures are effective antibacterial agents against *S. aureus* grown in planktonic culture and in biofilm. Moreover, this is the first report demonstrating the synergistic interaction between the *D. binata* extract and silver nanoparticles (AgNPs), which results in the spectacular enhancement of the observed bactericidal activity, while having no cytotoxic effects on human keratinocytes. Simultaneous use of these two agents in significantly reduced quantities produces the same effect, i.e. by killing 99.9% of bacteria in inoculum or eradicating the staphylococcal biofilm, as higher amounts of the agents used individually. Our data indicates that combining AgNPs with either the *D. binata* extract or with its pure compound (3-chloroplumbagin) may provide a safe and highly effective alternative to commonly used antibiotics, which are ineffective towards the antibiotic-resistant *S. aureus*.

## Introduction

Introduction of antibiotics to medicine has nearly eliminated multiple species of pathogenic microorganisms. However, widespread misuse of these drugs led to the emergence of multiple drug-resistant bacterial species [1]. Mutations which promote survival in the presence of antibiotics and horizontal transfer of such mutations, give rise to antibiotic-resistant strains such as methicillin-resistant *Staphylococcus aureus* (MRSA). Due to antibiotic resistance, *S. aureus* strains are particularly difficult to eliminate and therefore pose a significant threat in nosocomial infections [2]. *S. aureus* are common Gram-positive bacteria which belong to the harmless natural microflora that inhabits intact and healthy human skin. However, the presence of wounds or certain changes in skin physiology may influence the bacteria to shift to their pathogenic state. The structure of biofilm formed by bacterial cells and polysaccharides contributes to drug resistance of *S. aureus*. This three-dimensional structure is very complex and virtually impenetrable to most compounds and therefore it is significantly more resistant to commonly applied antibacterial therapeutics than planktonic cultures [3]. Antibiotic resistance of *S. aureus* strains is an important issue not only regarding drugs such as β-lactams, but also glycopeptides, aminoglycosides, quinolones and oxazolidinones [4]. Considerable problems due to antibiotic resistance promote the development of novel antibacterial drugs. Search for more effective ways to treat MRSA infections stimulates the investigation of natural compounds as alternative treatment [5].

Silver nanoparticles (AgNPs) are three-dimensional structures up to 100 nm in diameter formed from silver ions reduced to Ag^o^ clusters which are then stabilized by coating ligands. The antimicrobial activity of AgNPs has already been demonstrated towards multiple fungi and bacteria species, regardless of their susceptibility or resistance to common drugs [6]. The antimicrobial properties of AgNPs are attributed to the direct effect that these particles have on the bacterial cell as well as to the activity of silver ions continuously released from their surface [7]. Antimicrobial activity of silver nanostructures is based on their ability to affect cellular components such as the cell wall, membrane, proteins and nucleic acids [6].

**Figure 1:**
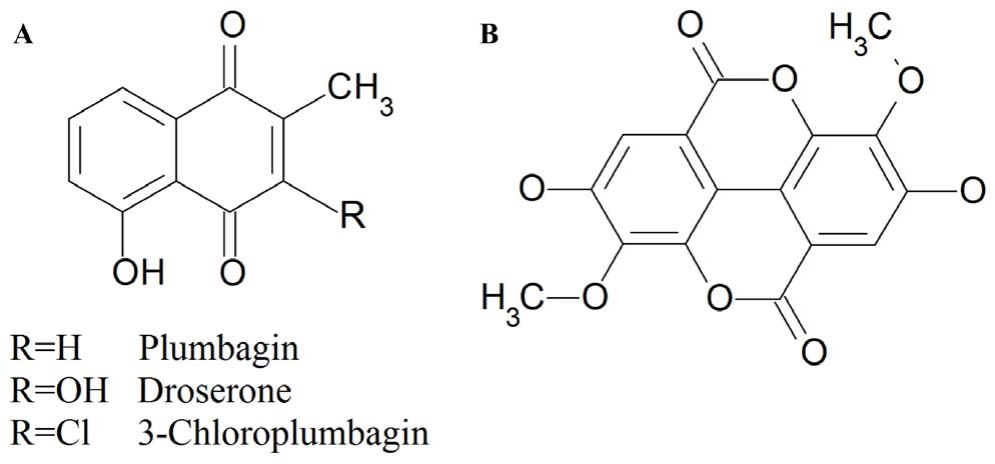
The structure of A) plumbagin, droserone, 3-chloroplumbagin and B) 3,3′-di-*O*-methylellagic acid.

The Drosera genus, native to Australia and New Zealand, includes multiple carnivorous species which possess substantial medicinal potential. Medicinal use of *Drosera* is convenient due to the simplicity of its cultivation *in vitro*. Our previous research regarding carnivorous plants has demonstrated that *D. binata* cultures are characterized by a high multiplication index in *in vitro* cultures. A single *D. binata* leaf explant is capable of producing 325 daughter plants within a year [8]. *Drosera* extracts owe their antimicrobial properties to secondary metabolites. Naphthoquinones, mainly plumbagin (Fig. 1a), are the main active compounds produced by *D. binata* tissues. The plants are also a source of flavonoids, ellagic acid and their glycoside and methyl derivatives [9]. It is crucial that plant extracts, unlike antibiotics, do not contribute to the emergence of resistant bacterial strains when used as antibacterial agents [10]. Various studies showed interactions between several secondary metabolites found in plant extracts, which allowed herbal drugs to be used in lower doses of active components [11]. Our research showed that one component of the *D. binata* extract has a synergistic interaction with AgNPs and that the three remaining components of the extract dramatically enhance this synergistic bactericidal activity. Moreover, use of the whole extract in antimicrobial experiments has high economic as well as practical value.

Nowadays the number of chemotherapy treatments based on the multidrug concept continuously increases. The basis of effective multidrug chemotherapy is the multi-target action of several drugs leading to the same therapeutic effect while significantly lowering the required active doses as well as the production cost [12]. Synergistic combinations of drugs are found to be extremely effective against antibiotic-resistant microorganisms. Therefore in this study we examined the antimicrobial potential of AgNPs and the *D. binata* extract against *S. aureus*. To the best of our knowledge this is the first report describing the efficiency of AgNPs combined with the *D. binata* extract in eliminating bacterial populations grown in planktonic and biofilm cultures.

## Results

### Antimicrobial activity

*S. aureus* strains were characterized by their susceptibility to antibiotics in order to evaluate their resistance against commercial drugs (Table 1). We determined the MBC values of the *D. binata* extract and AgNPs for planktonic cultures of *S. aureus*. The effective concentrations of the extract (16 µg DW/ml) and AgNPs (6.15 µg/ml) showed no significant differences between the tested strains. Only a single strain (MRSA 43300) required a slightly higher MBC for AgNPs, i.e. 9.25 µg/ml. Most importantly, the studied bacteria included strains which were either susceptible or resistant to antibiotics. As plumbagin is the main naphtoquinone presents in *D. binata* tissues we tested this pure compound as a control. Our study showed that 16 µg/ml of pure plumbagin had a bactericidal effect on *S. aureus*.

**Table 1:** Susceptibility of tested *Staphylococcus aureus* strains to antibiotics.

Strain of Staphylococcus aureus	Oxacillin		Ciprofloxacin		Vancomycin	
	MIC	MBC	MIC	MBC	MIC	MBC
	(µg/ml)					
ATCC 13420	0.25	0.25	0.25	0.5	4	32
MRSA 43300	2	128	1	2	2	4
703/k	16	128	0.5	64	2	128

MIC - Minimum Inhibitory Concentration.

MBC - Minimum Bactericidal Concentration.

**Figure 2:**
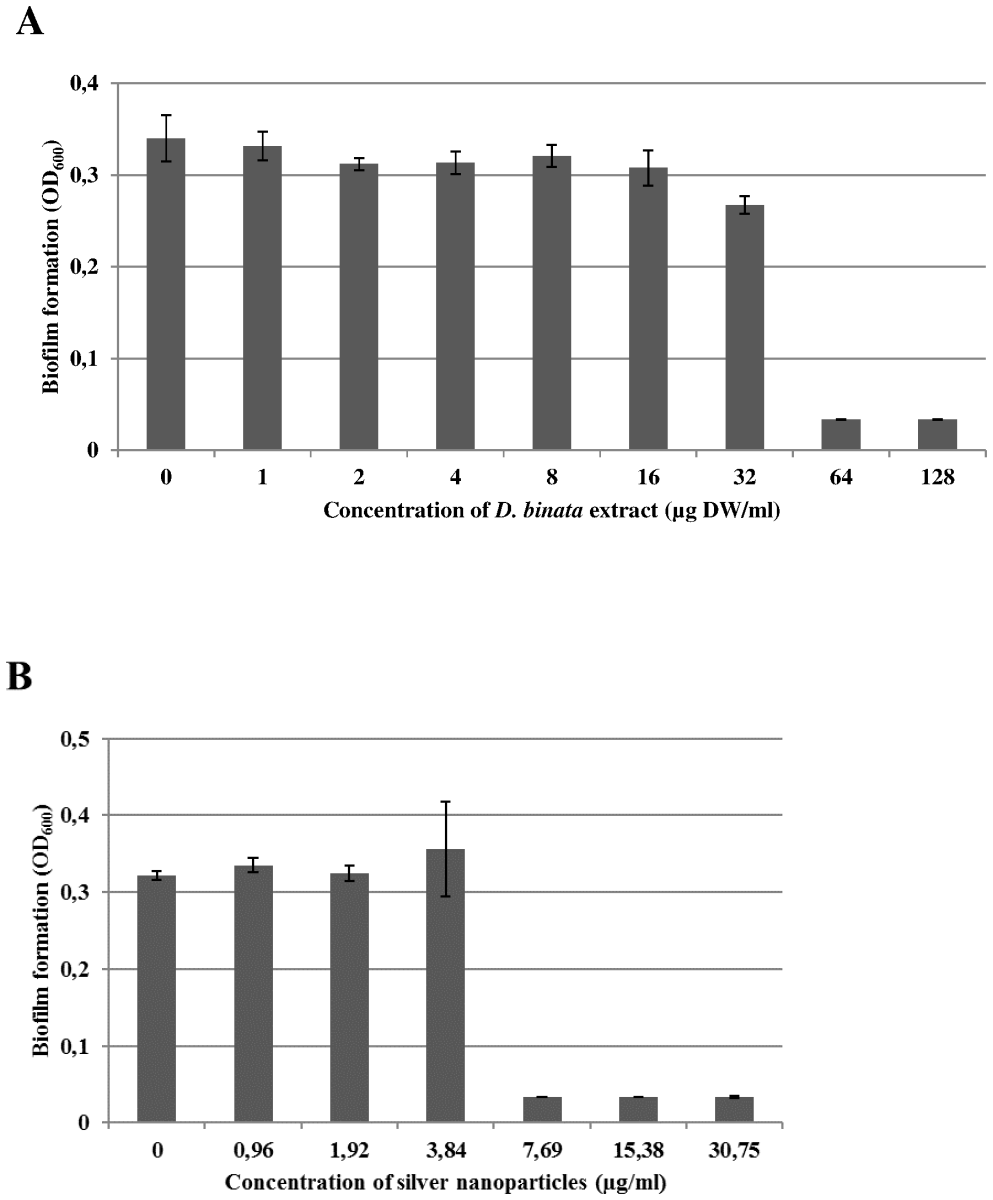
Eradication of *S. aureus* biofilm by A) the *D. binata* extract and by B) AgNPs. The experiment was conducted using the ATCC 13420 strain. The MBEC (Minimum Biofilm Eradication Concentration) obtained from the recovery plates after 24 h of incubation at 37°C is defined as the absorbance at 600 nm below the value of 0.1. DW – dry weight.

We also examined the effect of the plant extract (Fig. 2a) and the effect of AgNPs (Fig. 2b) on *S. aureus* ATCC 13420 biofilm. The number of cells growing in the staphylococcal peg biofilm was established as 4.51±0.58 log10 CFU peg^−1^. The Minimum Biofilm Eradication Concentration (MBEC) values obtained for AgNPs and the plant extracts were 7.69 µg/ml and 64 µg DW/ml, respectively (Fig. 2a, b).

### Composition of the *D. binata* extract

The analysis of the antibacterial activity exhibited by the obtained extracts suggests that plumbagin is merely one identified constituent of many possible antibacterial agents present in the plant tissues. This is supported by the fact that the amount of plumbagin in the plant tissues does not fully correlate with the antimicrobial properties of plant extracts. Our research shows that the MBC value for pure plumbagin is 16 µg/ml for all *S. aureus* strains. By using the *D. binata* extract we obtained MBC values of 16 µg DW/ml, which corresponded to 2.69 µg of pure plumbagin content in the extract (Tables 2, 3). We employed HPLC-DAD-ESI-MS to analyze the composition of the extract derived from *D. binata* cultures (Table 3). Plumbagin and 3,3′-di-*O*-methylellagic acid (Fig. 1b) were established as the main compounds present in chloroform extracts derived from the *D. binata* plant culture. Chromatography analysis additionally revealed the presence of droserone and 3-chloroplumbagin (Fig. 1A, Table 3). The carotenoid, lutein, was among the remaining compounds found in the examined chloroform extract, although its concentration was very low (Table 3).

**Table 2:** Minimum Bactericidal Concentrations (MBCs) of secondary metabolites present in *D. binata* tissues as well as their content within the MBC of the extract.

Secondary metabolites present in *D. binata* tissues	MBC of pure secondary metabolite	Bactericidal concentration of secondary metabolites within the MBC of *D. binata* extract
	µg/ml	µg/ml
Plumbagin	16	2.690
3,3′-di-*O*-methyl ellagic acid	>128	0.432
Droserone	>128	0.048
3-chloroplumbagin	32	0.011

**Table 3:** Chromatographic data of compounds identified by HPLC-DAD-ESI-MS method (t_R_, UV, [M-H]^−^/[M+H]^+^) in the chloroform extract from shoot culture of *D. binata.*

Peak	t_R_ (min)	UV (λ_max_ nm)	[M-H]^−^/[M+H]^+^ *m/z*	Compound	Content ± SD (µg/mg DW)
1	21.92	245, 374	329^−^/331^+^	3,3′-di-*O*-methylellagic acid	26.8±1.50
2	22.88	243sh, 284, 408	203^−^/205^+^	droserone	3.0±0.15
3	29.62	245sh, 265, 417	189^+^	plumbagin	166.7±8.20
4	39.22	278, 422	223^+^	3-chloroplumbagin	0.7±0.04
5	58.2	420, 445, 473	568^+^, 551^+^	lutein	Trace

DW - dry weight.

### Establishing the nature of the interaction between AgNPs and the *D. binata* extract

**Figure 3:**
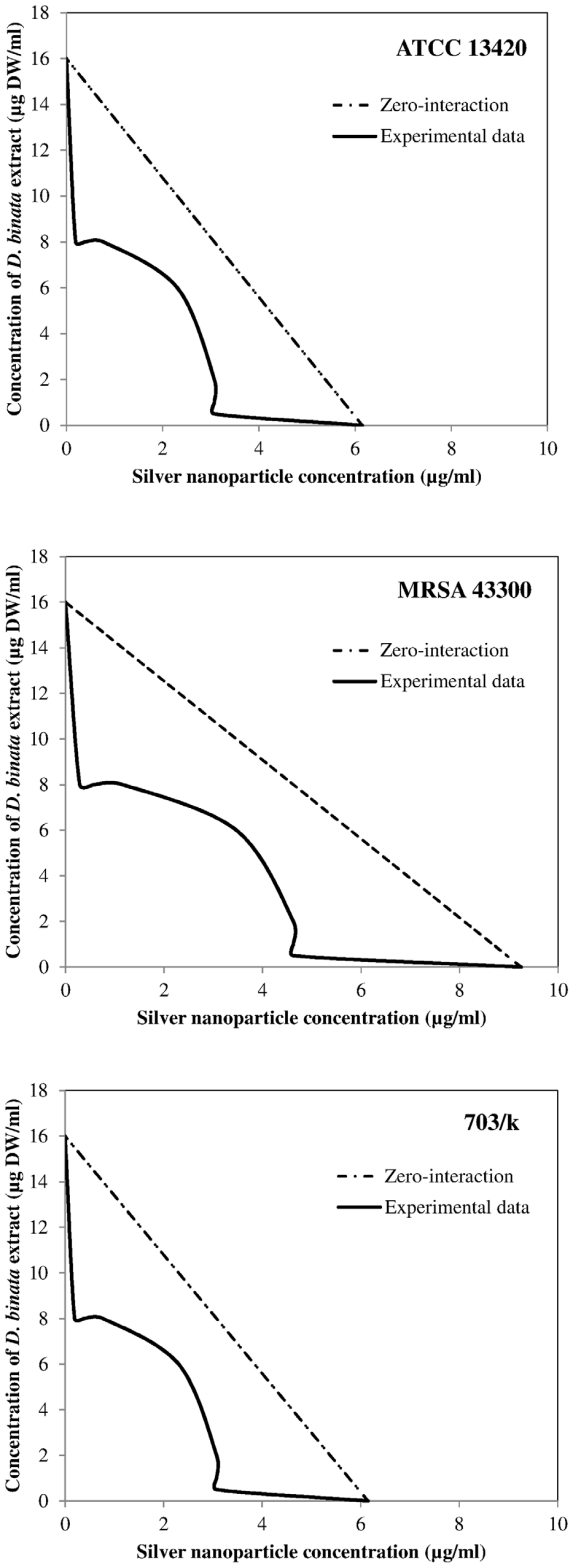
An isobologram depicting the bactericidal interaction between silver nanoparticles and either the extract derived from *D. binata* or pure 3-chloroplumbagin towards planktonic cultures of *S. aureus* strains. The Minimum Bactericidal Concentration was the measured effect.

**Figure 4:**
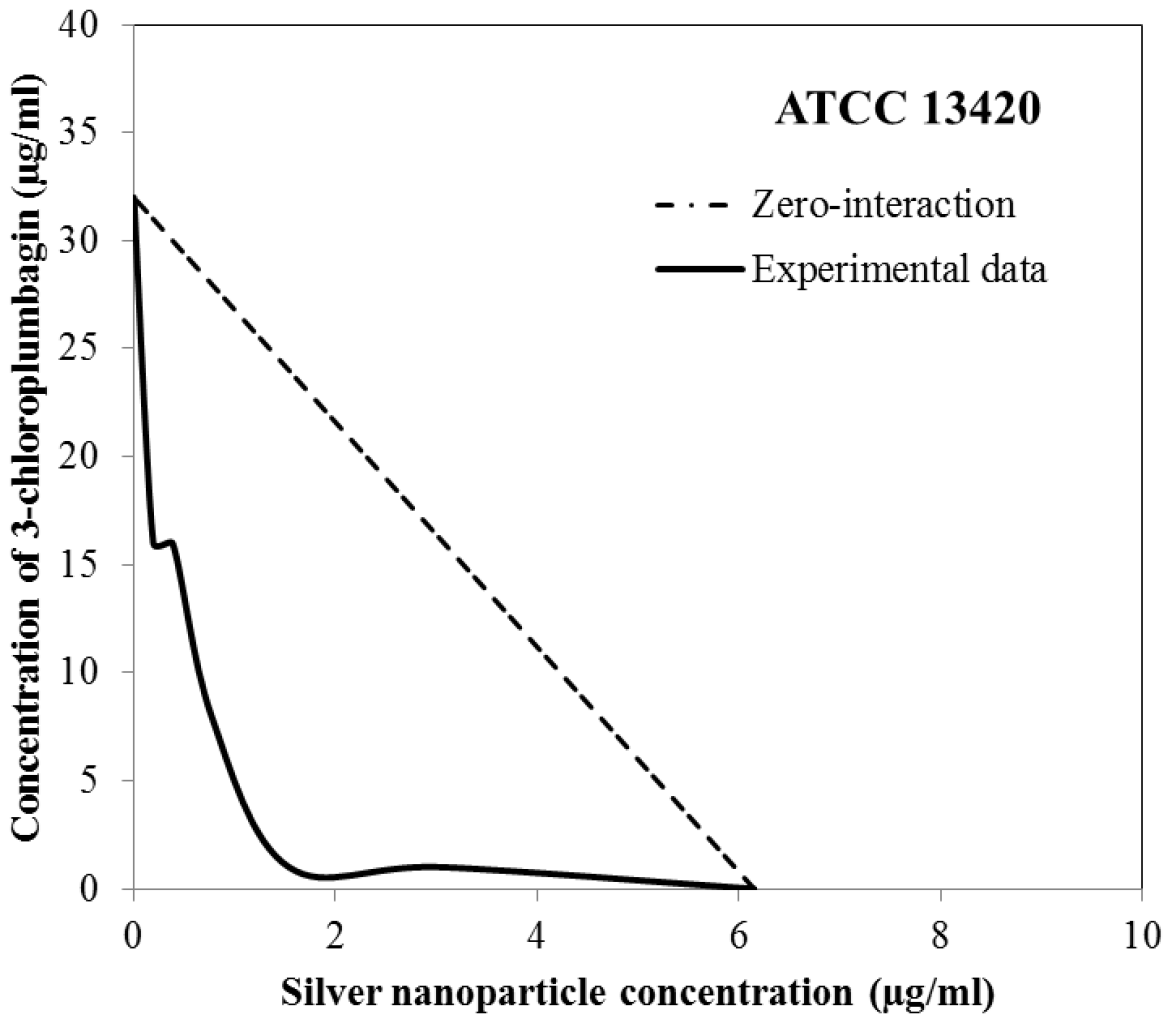
An isobologram depicting the bactericidal interaction between silver nanoparticles and pure 3-chloroplumbagin towards the planktonic culture of the *S. aureus* strain ATCC 13420. The Minimum Bactericidal Concentration was the measured effect.

With the use of the checkerboard titration technique, we investigated whether the antibacterial properties of AgNPs are enhanced by the addition of the plant extract. When the extract and AgNPs were used simultaneously against *S. aureus* the lowest value of the FBC index was 0.53 (Table 4). This value determines the synergistic nature of the bactericidal activity exhibited by AgNPs and the extract against all three *S. aureus* strains. As shown in the isobologram (Fig. 3) the synergistic mode of action between the agents occurs within defined concentration ranges. The same isobol pattern is observed regarding all 3 strains, with respect to differences in the initial concentrations of each agent. We also studied the possible interaction between AgNPs and pure compounds found in the extract, i.e. plumbagin, 3-chloroplumbagin, droserone and 3,3′-di-*O*-methylellagic acid. Based on our results, we singled out 3-chloroplumbagin as the compound which interacts synergistically with AgNPs. The FBC index obtained from this combination for the reference strain ATCC 13420 was as low as 0.28 (Table 5, Fig. 4).

**Table 4:** Results of the checkerboard assay for planktonic cultures of *S. aureus*: the FBC experimental values of AgNPs (A_f_ in µg per 1 ml) and the *D. binata* extract (B_f_ in µg of dry weight per 1 ml).

Combination		FBC index	Bactericidal concentrations of compounds after combination					
			ATCC 13420		MRSA 43300		703k	
A_f_/A_a_	B_f_/B_a_		A_f_	B_f_	A_f_	B_f_	A_f_	B_f_
0.500	0.030	0.530	3.075	0.5	4.630	0.5	3.075	0.5
0.500	0.060	0.560	3.075	1	4.630	1	3.075	1
0.500	0.125	0.625	3.075	2	4.630	2	3.075	2
0.375	0.375	0.750	2.310	6	3.470	6	2.310	6
0.125	0.500	0.625	0.770	8	1.160	8	0.770	8
0.062	0.500	0.560	0.380	8	0.580	8	0.380	8
0.031	0.500	0.530	0.190	8	0.290	8	0.190	8

A_f_/A_a_ and B_f_/B_a_ – fraction of bactericidal concentration of AgNPs and *D. binata* extract, respectively. A_a_, B_a_ – MBC of AgNPs (in µg per 1 ml) and the *D. binata* extract (in µg of dry weight per 1 ml), respectively. The interaction established by the value of the FBC index is considered to be synergistic at <1.0, additive at 1.0 and antagonistic at >1.0.

**Table 5:** Results of the checkerboard assay for planktonic cultures of *S. aureus*: the FBC experimental values of AgNPs (A_f_ in µg per 1 ml) and the 3-chloroplumbagin (B_f_ in µg per 1 ml).

Combination		FBEC index	Bactericidal concentrations after combination	
A_f_/A_a_	B_f_/B_a_		A_f_	B_f_
0.500	0.030	0.530	3.075	1
0.250	0.030	0.280	1.540	1
0.125	0.250	0.375	0.770	8
0.060	0.500	0.506	0.380	16
0.030	0.500	0.503	0.190	16

A_f_/A_a_ and B_f_/B_a_ – fraction of bactericidal concentration of AgNPs and 3-chloroplumbagin, respectively. A_a_, B_a_ – MBC of AgNPs (in µg per 1 ml) and the 3-chloroplumbagin (in µg per 1 ml), respectively. The interaction established by the value of the FBC index is considered to be synergistic at <1.0, additive at 1.0 and antagonistic at >1.0.

**Figure 5:**
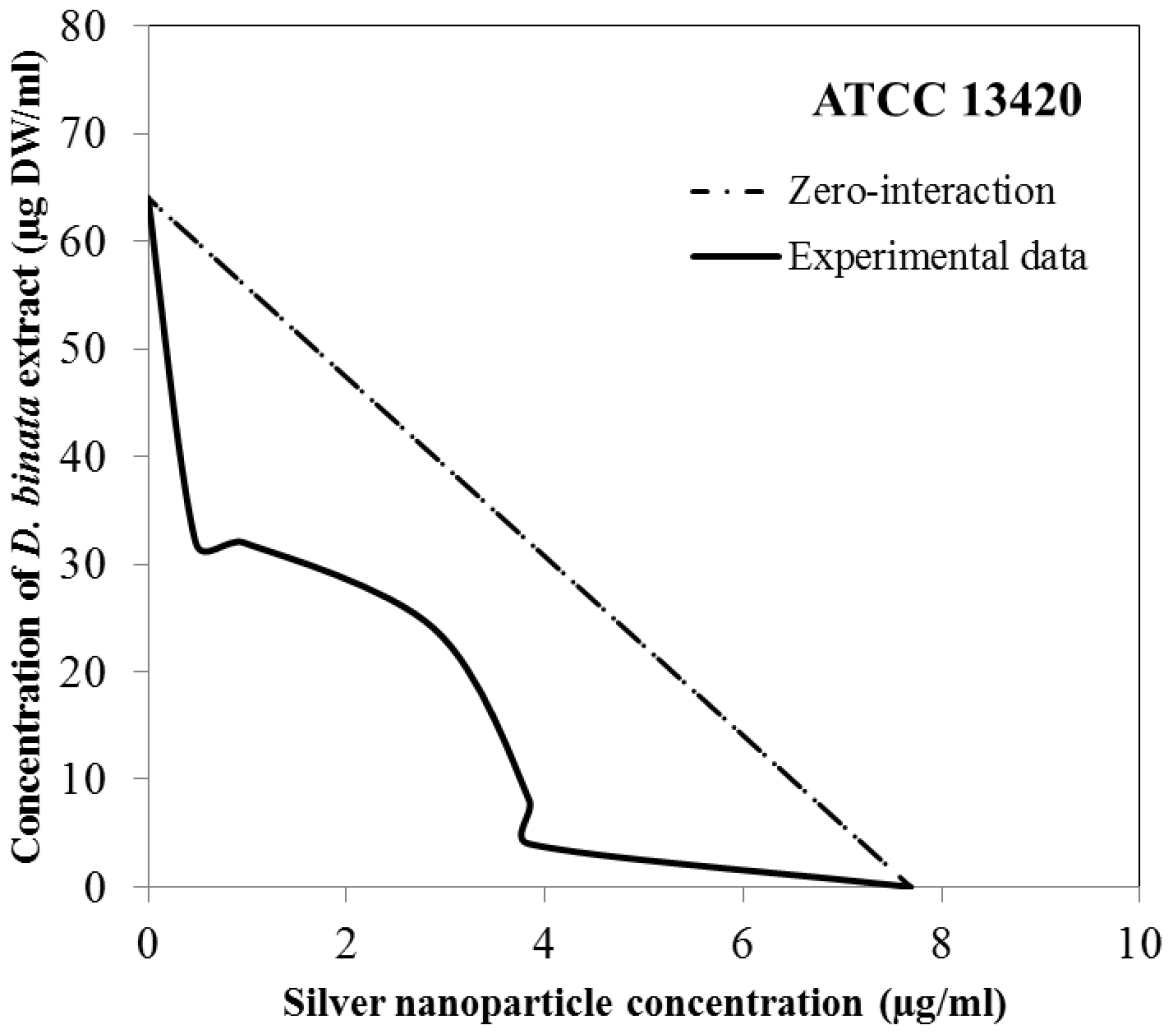
An isobologram depicting the bactericidal interaction between silver nanoparticles and the extract derived from *D. binata* towards biofilm formed by the *S. aureus* ATCC 13420 strain. The Minimum Biofilm Eradication Concentration was the measured effect.

We chose ATCC 13420 strain for tests investigating the antimicrobial potential of the extract combined with AgNPs towards bacteria grown in biofilm culture. The simultaneous application of these two antibacterial agents results in the significant reduction of their Fractional Biofilm Eradication Concentrations (FBECs). The lowest FBEC index obtained was 0.56 (Table 6). The FBEC index for biofilm is only slightly higher than the FBC index for planktonic cultures (0.53). According to the isobologram, combining the agents results in their synergistic activity towards staphylococcal biofilm (Fig. 5).

**Table 6:** Results of the checkerboard assay regarding *S. aureus* ATCC 13420 strain biofilm: the FBEC values of AgNPs (A_f_ in µg per 1 ml) and *D. binata* extract (B_f_ in µg of dry weight per 1 ml).

Combination		FBEC index	Biofilm eradication concentrations after combination	
A_f_/A_a_	B_f_/B_a_		A_f_	B_f_
0.500	0.060	0.560	3.840	4
0.500	0.125	0.625	3.840	8
0.375	0.375	0.750	2.880	24
0.125	0.500	0.625	0.960	32
0.060	0.500	0.560	0.480	32

A_f_/A_a_ and B_f_/B_a_ – fraction of bactericidal concentration of AgNPs and *D. binata* extract, respectively. A_a_, B_a_ – MBEC of AgNPs (in µg per 1 ml) and the *D. binata* extract (in µg of dry weight per 1 ml), respectively. The interaction established by the value of the FBEC index is considered to be synergistic at <1.0, additive at 1.0 and antagonistic at >1.0.

### Cytotoxic effects produced by antimicrobial agents towards human keratinocytes

**Figure 6:**
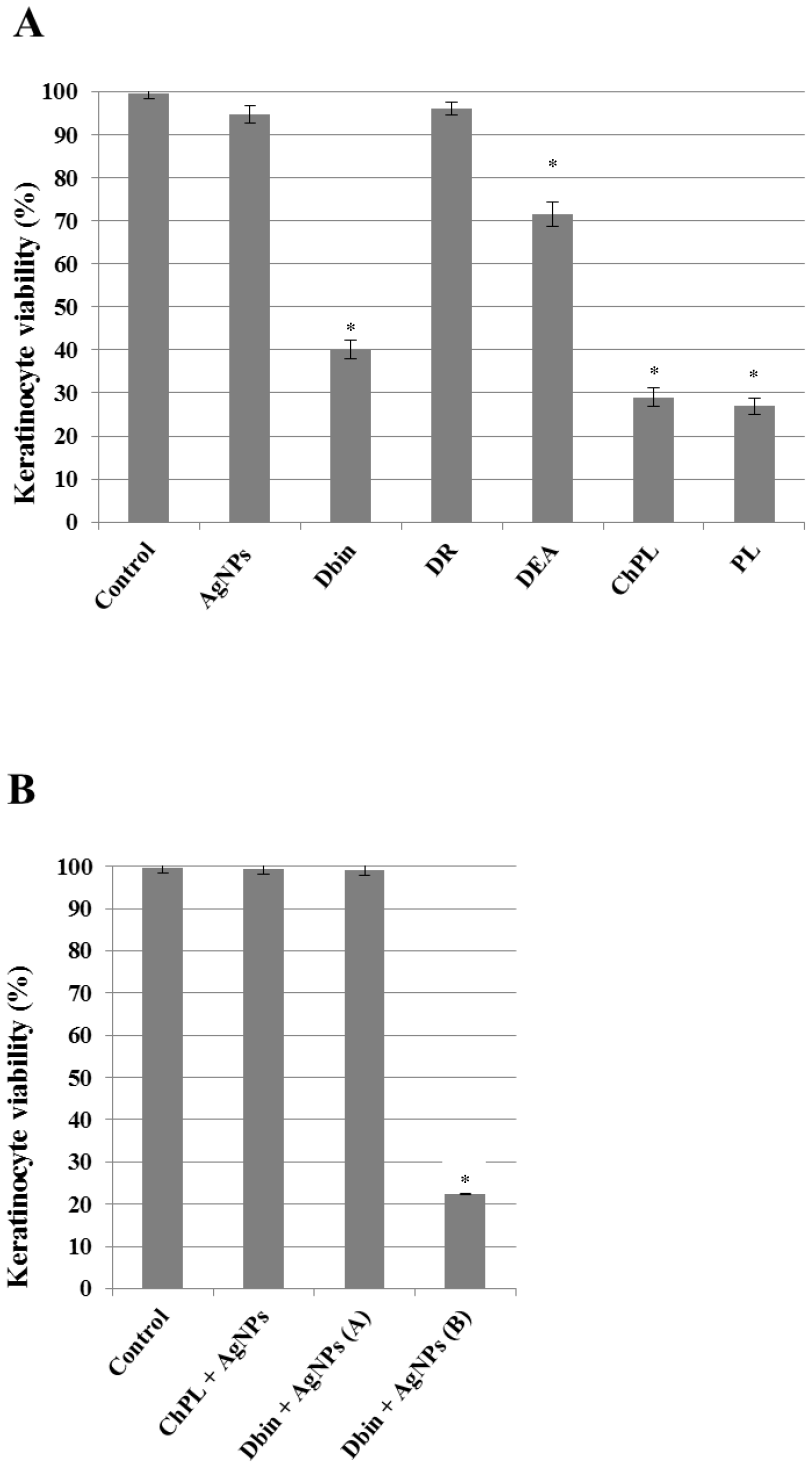
Cytotoxic effect of A) AgNPs, the *D. binata* extract and various secondary metabolites present in *D. binata* tissues applied individually on human keratinocytes [n = 3. Data were analyzed by one-way ANOVA with Tukey's post hoc tests; p≤0.05 (*) indicates differences between the control and 1-treated cells] and B) combinations of AgNPs with either the *D. binata* extract or with pure 3-chloroplumbagin towards human dermal keratinocytes. Toxicity was assessed after 24 h of incubation at 37°C in the dark. Each point is the mean of three experiments ± s.d. DR – droserone (32 µg/ml); AgNPs – silver nanoparticles (6.15 µg/ml); DEA – 3,3′-di-O-methylellagic acid (32 µg/ml); Dbin – chloroform extract from tissues of *D. binata* (16 µg DW/ml); ChPL – 3-chloroplumbagin (32 µg/ml); PL- plumbagin (16 µg/ml); Combinations: ChPL + AgNPs – 1 µg/ml+1.54 µg/ml, respectively; Dbin + AgNPs (A) – 0.5 µg DW/ml+3.075 µg/ml, respectively; Dbin + AgNPs (B) – 8 µg DW/ml+0.2 µg/ml, respectively [n = 3. Data were analyzed by one-way ANOVA with Tukey's post hoc tests, p≤0.05 (*)].

The synergistic bactericidal effect of AgNPs and *D. binata* extracts on *S. aureus* required examining their potential cytotoxicity towards healthy human cells. Therefore, human keratinocytes were treated with AgNPs and plant extracts and subsequently analyzed using the MTT assay in order to assess their viability. Keratinocytes were treated with *D. binata* extracts, pure compounds found in the extract and AgNPs, all of which were used in the lowest concentrations that exhibited significant bactericidal activity (MBC) (Table 2). The presence of 16 µg DW/ml of the *D. binata* extract resulted in a 60% decrease in cell viability (Fig. 6A). An even higher cytotoxic effect was observed in the presence of minimal bactericidal concentrations of plumbagin and 3-chloroplumbagin, where cell viability dropped 70% (Fig. 6A). AgNPs applied at a concentration of 6.15 µg/ml exhibited no cytotoxic effects (Fig. 6A). Most importantly, the following combinations: 0.5 µg DW/ml of *D. binata* with 3.075 µg/ml of AgNPs or 1 µg/ml of pure 3-chloroplumbagin with 1.54 µg/ml of AgNPs had no cytotoxic effect on human keratinocytes (Fig. 6B). Increasing the concentration of the extract to 8 µg DW/ml dramatically increased the cytotoxicity of the solution (Fig. 6B).

## Discussion

Before antibiotics entered mainstream health care, infectious diseases were treated by traditional medicine with the use of extracts from plant tissues, which are characterized by an enormous diversity of secondary metabolites. Nowadays, resistance of bacterial strains to conventional antibiotics reinforced the importance of developing new antimicrobial therapies in the field of phytotherapy. Likewise, metal nanostructures such as AgNPs, by having powerful antimicrobial properties and being the subject of constant development in nanotechnology, provide the solution to antibiotic resistance.

In this paper we present the first report which demonstrates that combining secondary metabolites produced by *D. binata* with AgNPs results in their strong antibacterial activity against *S. aureus* grown in planktonic as well as in biofilm *in vitro* cultures, whereas no cytotoxic effect is observed on human keratinocytes. This phenomenon is a result of the synergistic interaction which occurs between the two antimicrobial agents. Previously we have demonstrated that extracts obtained from several other *in vitro* cultured carnivorous plants possess antibacterial activity towards various pathogens in planktonic culture such as *Escherichia coli, Enterococcus faecalis, Klebsiella pneumoniae* and *S. aureus* [10]. The present study is the first report describing the efficiency of the *D. binata* extract itself in eliminating the dangerous human pathogen *S. aureus,* resulting however in the increased cytotoxicity on human keratinocytes. The antimicrobial effectiveness of the chloroform plant extract was similar towards all studied *S. aureus* strains, regardless of their resistance to antibiotics. However, a higher bactericidal concentration (MBEC 64 µg/ml) was required for *in vitro* cultured biofilm.

The biofilm structure is resistant to various antibacterial agents, therefore the observed reduced sensitivity of the *S. aureus* biofilm culture to the *D. binata* extract was likely to occur. This phenomenon is thought to result from poor penetration of compounds through the biofilm or from the presence of bacterial cells which are highly resistant to various agents [3]. It has been demonstrated that biofilm-forming *S. aureus* found in wounds is characterized by a three-fold higher resistance to the antibiotic mupirocin than planktonic *S. aureus* [13]. AgNPs, however, are equally effective regarding both types of cultures. Our studies on AgNPs show that their MBC values for planktonic culture and their MBEC values for biofilm cultured on pegs are very similar: 6.15 µg/ml and 7.69 µg/ml, respectively. It seems that the addition of AgNPs facilitates the penetration of the extract compounds through the biofilm structure. Studies performed by Kalishwaralal *et al.* [14] suggest that AgNPs are capable of blocking exopolysaccharide synthesis, which might explain their effectiveness in eradicating *S. aureus* biofilm.

Plumbagin is the main naphtoquinone present in *D. binata* tissues and it possesses significant antimicrobial activity towards Gram-positive bacteria [10]. Surprisingly, analysis of the composition of the *D. binata* chloroform extract by HPLC-DAD-ESI-MS revealed that the value of the minimum bactericidal concentration exhibited by plumbagin as one of the extract's constituents is actually lower than that of pure plumbagin. This suggested the presence of at least one unknown secondary metabolite compound in the chloroform extract derived from *D. binata* tissues that had a synergistic relationship with AgNPs, which resulted in the significantly increased antibacterial activity against *S. aureus*. As a result of further analysis we detected 3,3′-di-*O*-methyl ellagic acid and a very small amount of lutein as two other components of the extract. 3,3′-di-*O*-methyl ellagic acid was identified previously in the genus *Drosera* by Zehl et al. [9]. The antibacterial activity of ellagic acid is the subject of several publications [15], however there is little information about its methylated derivatives having similar activity. It was observed that the introduction of a methyl group to the phenolic compound moiety can increase its hydrophobicity and consequently its antimicrobial potential [15]. Surprisingly, our studies showed that pure 3,3′-di-*O*-methyl ellagic acid in the concentration of 128 µg/mL exhibited no antimicrobial activity towards *S. aureus*. Moreover, combining pure plumbagin with 3,3′-di-*O*-methyl ellagic acid did not result in any interaction between the two compounds. We observed a bactericidal effect of the mixture only when plumbagin was used in a concentration as high as its MBC [data not shown]. This data suggested that the *D. binata* extract contained at least one further compound, apart from plumbagin, 3,3′-di-*O*-methyl ellagic acid and lutein, which interacted in synergy with AgNPs, resulting in the observed strong antibacterial activity of the extract combined with AgNPs. It was certain that this substance was present in the extract in such minute quantity that it was near the lower limit of detection and could have been included in the background signal. Careful studies established droserone (3 µg/mg DW) and 3-chloroplumbagin (0.7 µg/mg DW) as the remaining compounds of the *D. binata* extract. This is the first report describing these secondary metabolites as constituents of the *D. binata* extract. The presence of droserone has previously been reported in tissues of *Dionaea muscipula* [16], *Drosera peltata* [17] and *Triphyophyllum peltatum* [18]. 3-chloroplumbagin has been found in tissues of *D. muscipula* [16], *Plumbago zeylanica* [19], *Drosera ramentacea* [20], *Diospyros maritime* BLUME [21], *Drosera intermedia* and *Drosera anglica* [22]. While droserone itself demonstrated no antimicrobial properties whatsoever (MBC>128 µg/ml), the use of 3-chloplumbagin in the concentration of 32 µg/ml had a mild antimicrobial effect towards *S. aureus*. Most surprisingly however, the simultaneous use of 3-chloplumbagin and AgNPs resulted in a spectacular enhancement of the observed antibacterial effect towards *S. aureus*. The same level of antibacterial activity was observed in concentrations as low as 1 µg/ml of 3-chloroplumbagin with 1.54 µg/ml of AgNPs, which demonstrated no cytotoxic effect on human keratinocytes. Multiple studies show that synergistic interactions are of vital importance in phytomedicines [11]. It has been stated before that chlorine-containing compounds, as well as some natural chlorine-containing compounds found in plants, possess strong biological activity [23]. Our results regarding 3-chloroplumbagin provide a good example for this observation.

However, the most important finding in this study was the observed synergy between the whole *D. binata* extract and AgNPs as a combined antibacterial agent against *S. aureus*. The simultaneous use of the two antibacterial agents resulted in their synergistic effect (FBC index 0.53; FBEC index 0.56) and the significant reduction of effective doses of these antimicrobials. Despite differences in the initial MBC values among the tested *S. aureus* strains in planktonic cultures, the shape of the isobole curves remained the same [Fig. 3]. Interestingly, the simultaneous use of the *D. binata* extract and AgNPs against *in vitro* cultured staphylococcal biofilm also resulted in similarly shaped isoboles [Fig. 5]. The obtained results demonstrate that these two combined agents (the extract and AgNPs or 3-chloroplumbagin and AgNPs) used in the above-mentioned proportions are equally effective towards both the planktonic and biofilm cultures of *S. aureus*. However, while pure 3-chloroplumbagin in the concentration of 1 µg/ml is required to produce a synergistic interaction with 1.54 µg/ml of AgNPs, the presence of merely 0.00035 µg/ml of 3-chloroplumbagin within the extract produces the same effect when 0.5 µg DW/ml of the extract is combined with 3.075 µg/ml of AgNPs. These results point to the fact that while 3-chloroplumbagin itself demonstrates a strong synergistic interaction with AgNPs, the three remaining components of the *D. binata* extract are actually indispensable for the full synergistic potential of this interaction to unfold. We must stress the importance of this finding due to the fact that creating an antimicrobial agent that is so potent and active in such extremely low concentrations is very rare in the antimicrobial drug industry, and can therefore be successfully used against *S. aureus*, while having no cytotoxic effects on human cells. No other tested combination (AgNPs with pure plumbagin; AgNPs with pure 3,3′-di-*O*-methyl ellagic acid; AgNPs with pure droserone, AgNPs with a mixture of these three compounds, with respect to their actual proportions found in the *D. binata* extract) exhibited any synergistic activity [data not shown].

The mechanism of the biological activity of plumbagin is not fully understood, however it is presumably similar to the mode of action attributed to naphthoquinones. This group of compounds covalently binds thiol groups present in proteins, chelates metal ions, which are required for the activity of various enzymes, intercalates to DNA and generates free oxygen radicals [24]. Furthermore, plumbagin is known for its ability to make resistant cells susceptible to antimicrobials through plasmid curing, which greatly contributes to fighting bacterial resistance [25]. The mechanism underlying the antimicrobial activity of AgNPs towards various cellular components such as the cell wall and membrane, proteins and nucleic acids has not been entirely elucidated. It is known that aggregates formed by AgNPs on the cell wall or membrane result in the formation of dents on the cell's surface and consequently lead to the release of cellular components and cell death [26]. Additionally, AgNPs enter the cell and release silver ions, which affect cellular compounds, i.e. proteins and nucleic acids, by interacting with multiple functional groups, including thiol, carboxyl, phosphorous, hydroxyl, imidazole and amine groups [26]. Regrettably, the available literature data does not explain the mechanism of 3-chloroplumbagin's activity towards bacterial cells. However, full understanding of this process will be the subject of our future research.

The multiple pathways through which the *D. binata* extract combined with AgNPs demonstrate their strong antibacterial activity gives prospects for new antimicrobial treatment which possesses multi-target antibacterial properties against resistant *S. aureus*. Therefore, further investigation of the exact mechanism of synergy between various secondary metabolites produced by *D. binata* and particles of silver is crucial. 

The combination of the *D. binata* extract and AgNPs has been found to be cytotoxic towards *in vitro* cultured human keratinocytes when a higher concentration of the extract was used (8 µg DW/ml). However, lowering the concentration of the extract to 0.5 µg DW/ml and combining it with 3,075 µg/ml AgNPs entirely eliminated this problem, while maintaining the strong antibacterial effect. Similarly, lowering the highly antibacterial, although cytotoxic, amount of pure 3-chloroplumbagin (32 µg/ml) to 1 µg/ml and combining it with 1.54 µg/ml AgNPs was very efficient in eliminating *S. aureus*, while demonstrating no cytotoxicity towards human keratinocytes. Since lutein is considered safe for humans and there is no evidence of droserone and 3,3′-di-O-methylellagic acid toxicity, the toxic effect of the *D. binata* extract seems to be attributed to the high concentration of plumbagin. As much as the above-mentioned cytotoxic activity observed *in vitro* is indisputable, studies involving the use of plumbagin *in vivo* on mice skin revealed no toxicity of plumbagin (5 µM) towards internal organs [27]. As antibiotic resistance of *S. aureus* is a serious problem in skin infections [28], the proposed new therapeutic agent consisting of the *D. binata* extract and AgNPs could be intended for further studies on novel therapies treating burn wound infections, impetigo or diabetic foot infections. Due to the fact that both alternative combinations are equally effective and exhibit no cytotoxic effects (AgNPs with the *D. binata* extract as well as AgNPs with pure 3-chloroplumbagin) this new therapeutic agent may be used as either alternative. However, the use of pure 3-chloroplumbagin raises concerns of an economic nature. This compound is present in plant tissues in very low quantities, therefore it is problematic to isolate significant amounts of this active substance from plant tissues. Furthermore, chemical synthesis of 3-chloroplumbagin is characterized by very low efficiency, i.e. 1 g of pure plumbagin used in the reaction produces merely 100 mg of pure 3-chloroplumbagin.

It is essential that the extracts derived from *in vitro* cultured *D. binata* tissues are characterized by a very stable quantitative and qualitative composition of secondary metabolites, which contributes to the repetitiveness of each subsequent experiment. Lack of growth factors in the *in vitro* culture limits the occurrence of somaclonal variation, thus avoiding possible changes in the composition of secondary metabolites.

Our results clearly show that when combined, AgNPs and the *D. binata* extract exhibit a strong bactericidal effect against both the planktonic and biofilm cultures of *S. aureus*. We demonstrate that the extract and AgNPs used simultaneously have the effect of a bactericidal agent which combines both agents' various mechanisms of antibacterial activity. Phytotheraphy based on the described *D. binata* extract and AgNPs has great potential in eliminating antibiotic resistant bacteria.

## Materials and Methods

### Plant tissue and extraction

*Drosera binata* tissues were obtained by micropropagation under *in vitro* conditions. *In vitro* cultures of *D. binata* were established using plants grown in peat-based soil [8]. Culture medium composed of half-strength Murashige Skooge [29] liquid medium (1/2 MS), 2% sucrose, 0.15% activated carbon, pH 5.5 were used for micropropagation. The plants were grown in 250-ml flasks containing 35 ml of medium in a growth chamber (temperature: 20–22°C; illumination: 25 µmol·m^−2^·s^−1^; 16 h/8 h light/dark cycle; rotary shaker set at 110 rpm, amplitude set to 9).

Following four weeks of *in vitro* culture samples were collected, washed in distilled water, dried using paper towels and then frozen (−20°C). Frozen plant tissue was ground in the presence of chloroform (40 ml of chloroform for each gram of tissue) using a mortar and pestle and the resulting sample was sonicated (XL–2020 Sonicator, Misonix, USA) at 35°C for 30 min. The extract was filtered (Whatman I paper) and the sample was dried under a stream of air at 40°C. The obtained dry matter was dissolved in chloroform (6 ml of chloroform/1 g of the initial fresh plant material). The *D. binata* chloroform extracts were dried, suspended in methanol and then filtered using the Millex filter (LG 0.2 µm LCR (PTFE) Merck Millipore) and stored at −20°C. The methanol extracts were dried under a stream of nitrogen and dry weight (DW) was determined using an analytical balance. The extract used later on in the antibacterial tests was dried and dissolved in dimethyl sulfoxide (DMSO).

### Analysis of extract composition

Individual compounds present in the chloroform extract from *D. binata* tissues were identified with the use of Shimadzu HPLC system consisting of CBM-20 system controller, steal wash pump LC-20AD, online degasser DGU-20A5, auto-sampler SIL 20AC, column termostat CT0-20AC, DAD and ESI-MS detectors. The compounds were separated using the Kinetex C18 column (100 mm×4.6 mm, 2.6 µm) (Phenomenex, USA). Column temperature was set at 20°C. The mobile phases consisted of 0.1% aqueous TFA solution (A) and 0.1% TFA solution in acetonitrile (B). The gradient elution programme was as follows: 0 min, 10% B; 50 min, 50% B; 60 min, 100% B; 75 min, 100% B. Flow rate was 1.0 ml/min. The injection volume was 1 µL. UV detection at 254 nm. ESI-MS parameters included: nebulizing gas flow 1.5 L/min, drying gas flow 16 L/min; detector voltage 1.6 kV, interface voltage 3.5 kV.

### Silver nanoparticles

AgNPs were provided by ProChimia Surfaces Co. (Poland) as water-soluble nanoparticles coated with the HS-(CH_2_)_11_-N(CH_3_)_3_^+^ ligand, 5.5 nm in diameter, characterized by a dispersity level of 15%. The initial stock concentration of AgNPs was 6.74×10^14^ NPs/ml which corresponds to 615 µg/ml (SPR maximum, λ_max_: 420–424 nm). We studied the antimicrobial activity of AgNPs within wide concentration ranges (0.48–61.5 µg/ml).

### Bacterial strains

We investigated the antimicrobial activity of *D. binata* extracts and AgNPs towards two multidrug-resistant clinical strains of *S. aureus* (MRSA 43300 and 703k) obtained from the Laboratory of Microbiology at the Provincial Hospital in Gdansk, Poland. The *S. aureus* ATCC 13420 strain was used as a reference strain in all experiments. Tested *S. aureus* strains were characterized by their susceptibility to antibiotics: oxacillin (Fluka Analytics), ciprofloxacin (AppliChem) and vancomycin (Sigma-Aldrich) accordingly to the CLSI standard broth microdilutions method [30]. All strains mentioned are deposited at the IFB, UG & MUG, Poland. Except for experiments concerning antimicrobial susceptibility testing bacteria were grown in Tryptic Soy Broth (TSB) or on Tryptic Soy Agar (TSA) (Oxoid, UK) at 37°C. Overnight bacterial cultures were diluted to obtain the initial inocula, equivalent to 0.5 McFarland turbidity standard (1.5−5×10^7^ Colony Forming Units [CFU]/ml) measured by densimeter (DensiMeter II, EMO, Brno).

### Bactericidal activity

The Minimum Bactericidal Concentrations (MBC) of studied antimicrobials were determined using the guidelines for reference broth microdilution method as described by the CLSI [31]. MBC values were defined as the lowest possible concentration of a given antibacterial agent which reduces the bacterial inoculum by 99.9% (3 log_10_ reduction) within 24 hours. The microtiter plates with antimicrobial solutions and bacterial suspension (5×10^5^ CFU/ml) were incubated for 24 h at 37°C. 10 µl of culture media from wells with no visible bacterial growth was subcultured on TSA medium (Oxoid, UK) in order to determine the MBC values. In addition to *D. binata* extract and AgNPs, we also tested the bactericidal activity of pure plumbagin (Sigma-Aldrich), 3,3′-di-*O*- methylellagic acid (Bertin Pharma), 3-chloroplumbagin and droserone (synthesized by dr E. Paluszkiewicz from the Technical University of Gdansk according to the method described by Razzakova *et al*. [32] and Behrman *et al.* [33] with several modifications). The compounds used for further testing were dissolved in DMSO. The final concentration of DMSO in the bacterial growth medium was below 0.5%, which is not toxic to bacterial cells. The *in vitro* bactericidal activity of antibiotics against *S. aureus* was evaluated for comparison purposes.

### Antibacterial activity against biofilm cultures

Biofilm obtained using the MBEC Assays (Innovotech) system consisting of lids equipped with pegs and 96-well plates according to the published protocol [34] was chosen as an *in vitro* model of staphylococcal biofilm. The biofilm was grown from an overnight bacterial culture diluted to 1.5×10^7^ CFU/ml. 150 µl of the bacterial suspension was added to each well, plates were covered with lids and incubated in 37°C on a rotary shaker set at 150 rpm for 24 h. Lids containing the newly formed biofilm were then gently washed in physiological saline (PS) and transferred to challenge plates supplied with serial dilutions of the studied antibacterial agents in TSB medium. After 24 h of incubation in 37°C the lids were washed in PS and sonicated for 5 min in recovery plates containing 200 µl of TSB medium for disrupting biofilm from the surface of the pegs into the medium. Microbial growth on untreated pegs was controlled by determination of viable cell counts (VCC) in appropriate wells of recovery plate after sonication. Next the recovery plates were incubated in 37°C for 24 h. The optical density of each well was established at 600 nm (Wallac 1420 Victor 2, Perkin Elmer). The Minimum Biofilm Eradication Concentration (MBEC) was defined as the lowest possible concentration of the antibacterial agent resulting in no visible growth of bacteria (OD_600_≤0.1). All experiments were performed in triplicate.

### Analysis of the interaction between AgNPs and the *D. binata* extract

In order to determine possible synergistic antimicrobial activity we analyzed various combinations of the plant extract, plumbagin, 3-chloroplumbagin, droserone, 3,3′-di-*O*- methylellagic acid and AgNPs using the checkerboard titration method [35]. We combined gradients of concentration dilutions of the two combined agents to specify their fractional bactericidal concentrations (FBC) or fractional biofilm eradication concentrations (FBEC). The mode of action of the two agents is expressed as the sum of their concentration fractions according to the equation: FBC (or FBEC) index  =  A_f_/A_a_ + B_f_/B_a,_ in which A_a_ and B_a_ are the MBC or MBEC values when AgNPs, extracts and secondary metabolite are used separately, whereas A_f_ and B_f_ are minimum bactericidal (or biofilm eradication) concentrations of AgNPs, extracts or secondary metabolite when they are used in combination. According to the equation, the nature of the interaction is identified as synergistic, additive, or antagonistic when the sum of the concentration fractions is: <1.0, 1.0 and >1.0, respectively. We used an isobologram to graphically illustrate the results of the checkerboard assay [36]. The graph is the geometric counterpart of the equation mentioned above. The shape of the obtained isobols indicates whether the interaction between the agents is synergistic (concave isobol), additive (straight isobol) or antagonistic (convex isobol).

### Cytotoxicity assay using eukaryotic cells

Human skin keratinocytes HaCaT (CLS order no. 300493) were cultured in Dulbecco's modified Eagle's medium supplemented with 10% fetal bovine serum, 2 mM glutamine, 100 units/ml penicillin and 100 µg/ml streptomycin. The cultures were incubated at 37°C in a humidified atmosphere containing 5% CO_2_. Human skin keratinocytes (5×10^4^) were seeded in 96-well plates and left to adhere overnight. The studied AgNPs (6.15 µg/ml), *D. binata* plant extracts (16 µg/ml), plumbagin (16 µg/ml), 3-chloroplumbagin (32 µg/ml), droserone (32 µg/ml) and 3,3′-di-*O*- methylellagic acid (32 µg/ml) were added to the medium separately as well as combined in a mixture corresponding with the lowest FBC index value: AgNPs (3.075 µg/ml) and plant extracts (0.5 µg DW/ml), AgNPs (0.2 µg/ml) and plant extracts (8 µg DW/ml) and AgNPs (1.54 µg/ml) and 3-chloroplumbagin (1 µg/ml). Cell viability was assessed by a standard 3-(4,5-dimethylthiazol-2-yl)-2,5-diphenyltetrazolium bromide (MTT) assay, in which MTT was the indicator of metabolically active cells. Due to the activity of mitochondrial enzymes in living cells, MTT is reduced to its pigmented product (formazan) which can be measured spectrophotometrically. MTT (0.5 mg/ml) was added following 24 hours of incubation with the studied agents, and the cells were incubated for 2 hours at 37°C. The cells were subsequently lysed with DMSO and the concentration of formazan was measured at 550 nm using a plate reader (Victor 2, 1420 multilabel counter).

### Statistical analysis

Values are expressed as means ± SD of at least three independent experiments. Differences between the control and 1-treated samples were analyzed by one-way ANOVA with Tukey's post hoc tests. A *p* value of ≤0.05 was considered as statistically significant in each experiment.
